# Fantastic proteins and where to find them – histones, in the nucleus and beyond

**DOI:** 10.1242/jcs.262071

**Published:** 2024-12-20

**Authors:** Johanna Grinat, Noah P. Shriever, Maria A. Christophorou

**Affiliations:** Epigenetics, Babraham Institute, Cambridge CB22 3AT, UK

**Keywords:** Chromatin, Evolution, Extracellular, Histones, Post-translational modifications, Signalling

## Abstract

Animal genomes are packaged into chromatin, a highly dynamic macromolecular structure of DNA and histone proteins organised into nucleosomes. This accommodates packaging of lengthy genomic sequences within the physical confines of the nucleus while also enabling precise regulation of access to genetic information. However, histones existed before chromatin and have lesser-known functions beyond genome regulation. Most notably, histones are potent antimicrobial agents, and the release of chromatin to the extracellular space is a defence mechanism nearly as ancient and widespread as chromatin itself. Histone sequences have changed very little throughout evolution, suggesting the possibility that some of their ‘non-canonical’ functions are at play in parallel or in concert with their genome regulatory functions. In this Review, we take an evolutionary perspective of histone, nuclear chromatin and extracellular chromatin biology and describe the known extranuclear and extracellular functions of histones. We detail molecular mechanisms of chromatin release and extracellular chromatin sensing, and we discuss their roles in physiology and disease. Finally, we present evidence and give a perspective on the potential of extracellular histones to act as bioactive, cell modulatory factors.

## Teaser

**Summary:** We discuss the evolution and mechanisms of chromatin externalisation and (patho)physiological functions of extracellular histones, including a perspective on their potential to act as bioactive, cell modulatory factors.

## Introduction

Inside each animal cell, ∼2 m of DNA (Alberts et al., 2002) must be accommodated within a microscopic nucleus. Prokaryotes use supercoiling to organise their DNA, whereas in eukaryotic cells this challenge is solved by histones, which package DNA into chromatin. It is hypothesised that histones were initially selected to limit access to DNA by viruses or transposons (Madhani, 2013), or to protect it from environmental damage; however, the capacity for gene regulation afforded to cells by the association of DNA with histones has shaped eukaryotic evolution and multicellularity.

Nucleosome occupancy governs access of epigenetic and transcriptional regulators to DNA and thus must be modulated. Enzymatic and non-enzymatic post-translational modifications (PTMs) to the N-terminal histone tail sequences in particular (Bannister and Kouzarides, 2011) modulate DNA accessibility, ultimately determining gene expression outcomes and cell responses (Jenuwein and Allis, 2001). The histone code hypothesis, which posits that the combination of PTMs present on histones acts as a code that determines the binding of epigenetic regulators and their downstream effects, is increasingly gaining support (Voigt et al., 2012; Lukauskas et al., 2024).

**Fig. 1.**
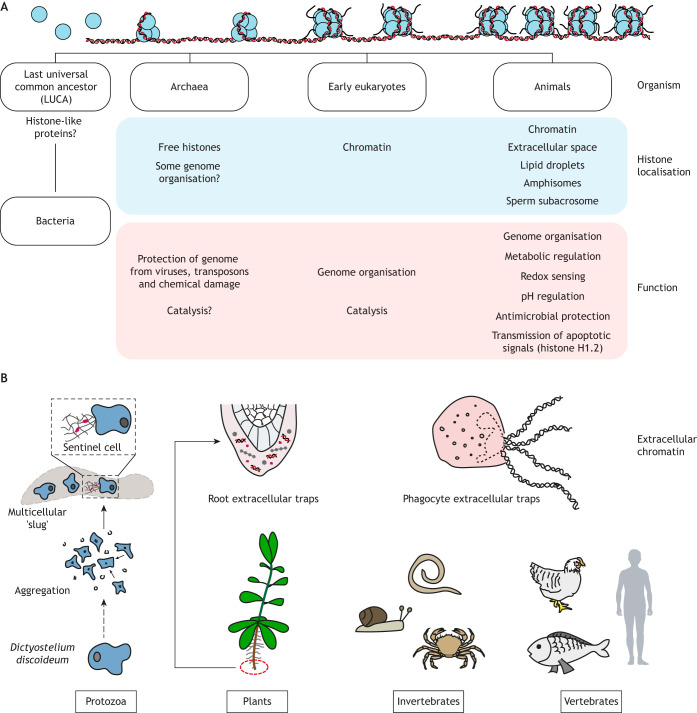
**Evolution of histones and chromatin.** (A) A timeline of the evolution of histones, their complexing with chromatin, and other known or hypothesised functions. Histone proteins are represented as blue spheres. Archaeal histones mostly lack N-terminal tails and form a looser chromatin structure, whereas compaction is increased in simple and higher eukaryotes. (B) Modes of chromatin release throughout the tree of life. The slime mould *Dictyostelium discoideum* releases chromatin via ETosis only while in a multicellular ‘slug’ phase. Plants release chromatin via root extracellular traps for defence against pathogens, whereas animal cells carry out ETosis in many contexts, such as immune or inflammatory responses or tissue repair.

The high conservation of histone sequences, including PTM sites, is indicative of their fundamental importance. However, histones precede the evolution of chromatin (Boxes 1 and 2; Fig. 1A), and current evidence shows that they can have extranuclear or extracellular localisations and functions independent of their roles in genome organisation and regulation. For example, histones have potent antimicrobial properties, and many organisms release their chromatin to the extracellular space as a form of defence against pathogens (Fig. 1B). This phenomenon is best studied in the context of neutrophil extracellular trap (NET) formation (NETosis).

**Box 1. Histones through the ages**

Histones are truly ancient proteins (Fig. 1A). The identification of archaeal homologues to eukaryotic histones suggests that they emerged before the evolutionary split between archaea and eukaryotes (Sandman et al., 1990; Sandman and Reeve, 2006). In certain classes of DNA viruses, fused histone pairs (expressed by genes encoding histone ‘doublets’ with linked histone fold domains) assemble to form nucleosomes that are virtually identical to eukaryotic counterparts (Valencia-Sánchez et al., 2021; Yutin et al., 2009). Histone homologues are also present in certain bacteria (Hocher et al., 2023), suggesting that histone-like proteins originated in the last universal common ancestor (LUCA) (Alva and Lupas, 2019).

The core histones H3, H4, H2A and H2B are very highly conserved, with their sequence differing minimally between highly unrelated species (Waterborg, 2012). It is worth pointing out, however, that histones have evolved to accommodate larger genome sizes – for example, through the acquisition of arginine residues (Macadangdang et al., 2014). Additionally, the sequences of histone variants, which have specialised functions in genome biology (Martire and Banaszynski, 2020; Yuan and Zhu, 2012), continue to evolve (Raman et al., 2022). These variants exhibit tissue-specific expression and might achieve atypical genome packaging, such as that required during development (Raman et al., 2022).

**Box 2. The evolution of chromatin**

Although genome size has increased over the course of evolution, the nucleus has not undergone similar expansion, creating the need for compacted genomes, which has been achieved by the complexing of DNA with histones (Hajheidari et al., 2019) (Fig. 1A). Some archaeal histone paralogues have DNA organisational properties (Stevens et al., 2020), suggesting that chromatin organisation originated in archaea (Bowerman et al., 2021). Increased compaction necessitated increased regulation of DNA accessibility. Archaeal histones possess a limited number of modifications, but the eukaryotic histone PTM repertoire has greatly expanded, accompanied by the evolution of histone PTMs and PTM ‘readers’ (Grau-Bové et al., 2022).

The fossil record suggests that the early origins of prokaryotic transcription factor-based gene expression processes appeared 1–2 billion years earlier than nucleosome-based modulation of gene expression in eukaryotes (Talbert et al., 2019). Whereas the complexing of DNA with histones is a property of archaea and eukaryotes, many of the histone-modifying enzymes that regulate eukaryotic transcription first emerged in prokaryotes. The last eukaryotic common ancestor (LECA) likely possessed many of the features of eukaryotic chromatin regulation, acquired from bacteria and co-opted for gene regulation in nucleosome-compacted chromatin (Talbert et al., 2019). Eukaryotic chromatin therefore has archaeal roots, but its regulation through histone PTMs is largely a eukaryotic innovation (Grau-Bové et al., 2022). Through posing a barrier to DNA accessibility, chromatin compaction provided a platform for cell differentiation, thus contributing to the morphological and functional complexity of multicellular organisms (Hajheidari et al., 2019; Talbert et al., 2019). The association of DNA with histones can therefore be considered a root event for eukaryotic evolution.

NETosis was first documented in 2004, when it was discovered that, under pathogenic challenge, neutrophils expel their cellular contents into the extracellular space (Brinkmann et al., 2004). This seminal study provided a formative advance that placed the antibacterial properties of histones into broader context and reconciled the seemingly disparate nuclear and non-nuclear histone functions that were known up to that point. It is now apparent that extracellular chromatin trap (ET) formation (ETosis) is an evolutionarily conserved, primordial antimicrobial process present across the tree of life (Box 3).

**Box 3. The evolution of extracellular chromatin**

ETs are present in protozoa and in invertebrate and vertebrate animals (Brogden et al., 2012; Chuammitri et al., 2009; Ramos-Martínez et al., 2021; Robb et al., 2014; Singh et al., 2023; Vandepas et al., 2024; Zhao et al., 2022), whereas plants release root extracellular traps (RETs) as part of an innate immune defence mechanism (Shirakawa et al., 2023; Wen et al., 2009) (Fig. 1B). Nearly all DNA release events involve histones and NADPH oxidase (NOX)-dependent mechanisms (Ramos-Martínez et al., 2021). Although RET activation is associated with reactive oxygen species and upregulation of a NOX-like gene, it is not yet clear whether the underlying molecular mechanism is similar to that of ETs. However, RETs and ETs, including NETs, share many molecular, structural and functional characteristics (Ramos-Martínez et al., 2021).

ETs are thought to pre-date the evolution of the coelom, the fluid-filled cavity containing the internal organs (Robb et al., 2014). Evolutionary analyses suggest that ETs have multiple, homoplastic origins (Ramos-Martínez et al., 2021). Unicellular organisms are unlikely to undergo ETosis, as this involves cell death and is detrimental to their survival. ETs are therefore hypothesised to be linked to the evolution of multicellularity, which allows differentiation, specialisation and sacrifice of some cells for defence (Ramos-Martínez et al., 2021). It is notable that *Dictyostelium discoideum* (a cellular slime mould)*,* which has a life cycle that includes a unicellular stage and a multicellular ‘slug’ stage, only undergoes ETosis in the multicellular, aggregative phase (Zhang et al., 2016), using Sentinel cells that undergo NOX-dependent DNA release when exposed to bacteria (Fig. 1B). This behaviour is suggested to be a precursor of ETosis (Zhang and Soldati, 2016).

Histones have additional functions – for example, in regulating cellular metabolism – and are even capable of catalysis (Ye et al., 2017; Attar et al., 2020; Cheng and Kurdistani, 2022). Various mechanisms exist for chromatin externalisation from dying or living cells. Once outside the cell, histones are not only antimicrobial but are associated with cytotoxicity and pathological inflammation (Ganapathy and Shyamala Devi, 2005; Xu et al., 2009; Collier et al., 2019; Silvestre-Roig et al., 2019). However, the roles of extracellular chromatin components and sensing pathways are more nuanced and potentially have positive impacts on tissue biology. Emerging evidence suggests that histones can associate with cell surface receptors and initiate downstream signalling that modulates cellular responses (Xu et al., 2011; Ibañez-Cabellos et al., 2018; Tsourouktsoglou et al., 2020; Wilson et al., 2022).

In this Review, we overview the current understanding of histone, chromatin and extracellular chromatin evolution and discuss the known functions of histones beyond genome regulation. We summarise the known molecular mechanisms of chromatin release and sensing, as well as the pathophysiological roles of extranuclear and extracellular chromatin components. Finally, we discuss gaps in the current knowledge and conclude by giving our perspective on the potential of extracellular histones to function as bioactive molecules that impact cell fates and functions.

## ‘Non-canonical’ cellular locations and functions of histones – beyond genome organisation

The existence of histones prior to chromatin, in combination with their high sequence conservation (Box 1; Fig. 1A), suggests that some of their original functions might also be at play in animal cells. We discuss the known ‘non-canonical’ functions of histones, including theories on ancestral histone functions and how these were co-opted into genome organisation (Boxes 1 and 2).

### Histones as antimicrobial agents

Histones and protamines (the germ cell-specific versions of histones) have long been known to possess antimicrobial properties (Hirsch, 1958; Miller et al., 1942; Reiner et al., 1942). Core and linker histones are effective against both gram-positive and gram-negative bacteria, and they act by destroying the bacterial cell wall or by binding to bacterial DNA and inhibiting transcription (Doolin et al., 2020; Hirsch, 1958; Hoeksema et al., 2016; Richards et al., 2001; Tagai et al., 2011). Free histones are more effective than nucleosomes at killing bacteria (Li et al., 2010).

Extracellular histones are found in immune-privileged animal tissues. In the gut, extracellular linker histone H1, potentially originating from dying epithelial cells, might provide protection against pathogens (Rose et al., 1998). Histone H1 is also present in cytosolic lipid droplets and is released in response to lipopolysaccharide (LPS) or lipoteichoic acid, thereby mediating antibacterial immunity and providing a significant survival advantage in *Drosophila* embryos (Anand et al., 2012; Cermelli et al., 2006). Extranuclear histones are also found on the epithelial surface of human placental syncytiotrophoblasts and amnion cells (Kim et al., 2002); the latter constitutively release histones H2A and H2B, which promote the antibacterial activity of human amniotic fluid, protecting the placenta and the foetus. Histone H4 released by human sebocytes (sebum-producing epithelial skin cells) is one of the major antimicrobial agents in sebaceous secretions, which defend against skin pathogens (Lee et al., 2009). The fact that different histones operate as extracellular antibacterial agents in different tissue contexts suggests the existence of release mechanisms that are more selective than total chromatin release. However, it is possible that the apparent selectivity is due to technical aspects of these experiments, such as detection methods and limits.

N-terminal histone cleavage products, such as parasin I, hipposin I and buforins I and II, have been identified in non-mammalian aquatic vertebrates and shown to have antibacterial activities (Kawasaki and Iwamuro, 2008). Studies in the Asiatic toad (*Bufo bufo gargarizans*) show that buforin I is produced by proteolytic cleavage of unacetylated histone H2A present in the cytoplasmic granules of gastric gland cells (Kim et al., 2000). The potent antibacterial properties of such naturally occurring histone-derived peptides have inspired the design of novel antimicrobial peptides that could act as alternatives to conventional antibiotics (Roshanak et al., 2021; Tsao et al., 2009).

### Histone complexes as enzymes

Recently, the remarkable discovery was made that the histone H3–H4 tetramer is capable of catalysis. Recombinant H3–H4 tetramers have a structurally predicted copper (Cu^2+^) binding site, and biochemical and mutagenesis experiments have shown that H3–H4 tetramers bind Cu^2+^ and catalyse its reduction to Cu^1+^ (Attar et al., 2020). Mutation of the Cu^2+^ binding site in the H3–H4 tetramer alters intracellular Cu^1+^ abundance and copper-dependent cellular functions in yeast (Attar et al., 2020). The authors posit that the original function of histones was to facilitate intracellular production of Cu^1+^ and that this might have helped organisms adapt to the global oxygenation event, which significantly reduced the concentrations of reduced metal forms on Earth (Lyons et al., 2014).

Native nucleosomes purified from *Saccharomyces cerevisiae* also possess oxidoreductase activity, suggesting that chromatin can act as a ‘metabolic organelle’ (Vogelauer et al., 2023 preprint). Notably, this activity requires Zn^2+^, which is also a co-factor for transcriptional and epigenetic regulators such as zinc finger transcription factors and histone deacetylases (Cassandri et al., 2017; Porter and Christianson, 2019; Vogelauer et al., 2023 preprint). This raises the intriguing possibility that some ancestral functions of histones take place alongside their genome regulatory roles.

### Histones as metabolic regulators

Histone methylation can modulate the capacity of eukaryotic cells to carry out methylation of other substrates (such as proteins or nucleic acids). In yeast cells that lack the phospholipid phosphatidylethanolamine, a major consumer of *S*-adenosylmethionine (SAM), histones become hypermethylated and act as methyl sinks that enable the conversion of SAM to *S*-adenosylhomocysteine, thereby promoting SAM homeostasis, sulfur metabolism and essential methylation reactions (Ye et al., 2017). Multi-omic analyses have demonstrated that histone methylation is inversely correlated with the presence of well-established metabolic sinks in human cells (Perez and Sarkies, 2023). Notably, although these associations are present on a genome-wide level, they do not correlate with transcriptional changes, suggesting that the metabolic function of histones is independent of their roles in regulating transcription.

The notion of chromatin as a ‘metabolic organelle’ is strengthened by discoveries that carbon starvation and subsequent depletion of acetyl coenzyme A (AcCoA) – a major precursor of cellular energy generation, biosynthesis and protein acetylation – leads to a reallocation of acetyl groups on histones (Cheng and Kurdistani, 2022). The redistribution of histone acetyl marks leads to reprogramming of gene expression that mediates the replenishment of AcCoA (Hsieh et al., 2022). Under conditions of increased metabolic demand, hyperacetylated histones can serve as a carbon source for lipid biosynthesis (Charidemou et al., 2024). Furthermore, chromatin can regulate intracellular pH levels. Reduction in intracellular pH leads to a global decrease and redistribution of histone acetylation (McBrian et al., 2013) and release of acetate anions, which helps to stabilise intracellular pH.

Thus, a picture is beginning to emerge of chromatin as a modulator of cellular physiology, beyond its roles in DNA regulation. The chromatin functions described above likely influence cell transformation and cancer development, where perturbations in cell metabolism are operative (Kurdistani, 2014).

### Cytoplasmic histone H1 as a messenger of apoptosis

Histone H1 is evicted from chromatin and released to the cytoplasm upon genotoxic damage (Konishi et al., 2003). Cytoplasmic histone H1.2 induces cytochrome *c* release and initiates apoptosis, thereby acting as a signal communicating the presence of DNA damage to the mitochondrion. Strikingly, histone H1.2 uniquely possesses this property, suggesting a specific signalling mechanism beyond mere release of histones due to DNA damage. Based on these findings, a histone H1-derived peptide has been used as a probe for imaging apoptosis in tumour cells (Wang et al., 2010).

### Extranuclear histones in sperm

In sperm, a histone H2B variant, subacrosomal H2B (subH2B or H2B.L), localises to a perinuclear structure called the subacrosome, which is located between the nucleus and the acrosome (Aul and Oko, 2001; Govin et al., 2007). The acrosome is a vesicular organelle that develops during sperm maturation and contains enzymes that facilitate penetration of the sperm into the zona pellucida of the egg cell (Berruti and Paiardi, 2011). The function of subH2B in this context is unknown, and it is unclear whether it is released along with the contents of the acrosome during fertilisation. A related histone variant, histone H2B.N, lacks the C-terminal acidic patch that mediates chromatin interactions (Raman et al., 2022). These findings raise the possibility that certain histone H2B variants have non-nucleosomal functions (Raman et al., 2022). It will be exciting to understand whether differing amino acid sequences of histone variants mediate their participation in complexes other than the nucleosome.

## Mechanisms of chromatin release in immunity, development and homeostasis

**Fig. 2.**
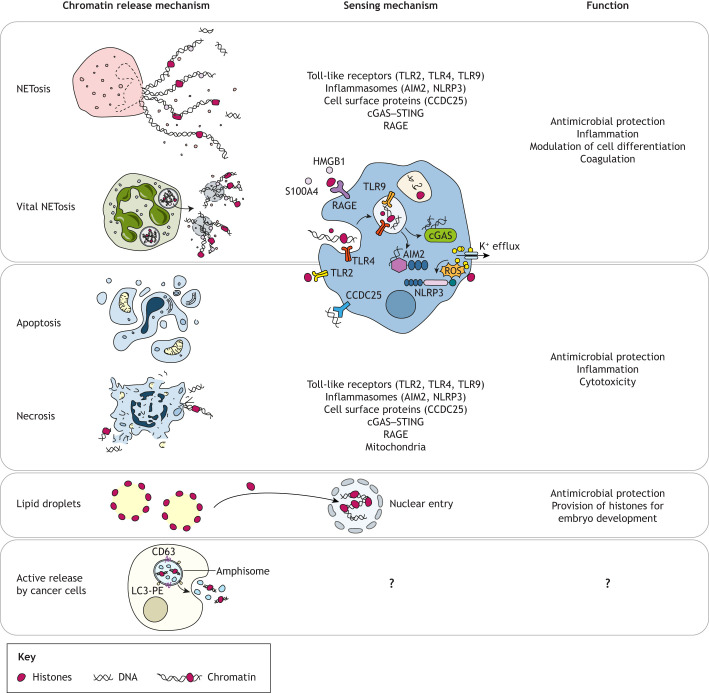
**Mechanisms of chromatin release and sensing in animals.** There are several known mechanisms of chromatin release, modes of chromatin sensing and functions of extranuclear or extracellular chromatin in higher animals. Chromatin can be actively released via suicidal or vital NETosis to modulate immune, inflammatory and repair processes. Chromatin is also released as a consequence of apoptosis and necrosis. In healthy cells, extranuclear histones can be stored in lipid droplets, which potentially serves gene regulatory or protective purposes in specific contexts such as embryonic development. Cancer cells can also actively release chromatin from amphisomes or within exomeres, although the function of these processes remains unclear. LC3-PE, phosphatidylethanolamine-conjugated microtubule-associated protein light chain 3B (MAP1LC3B).

Below, we discuss modes of chromatin and histone release by both dying and viable cells (Fig. 2).

### Suicidal and vital NETosis

The prototypical chromatin-releasing cells in vertebrates are neutrophils, which employ NETosis to kill invading pathogens. Different NETosis pathways can be activated depending on the stimulus (Kenny et al., 2017). Here, we focus on NADPH oxidase (NOX)-dependent and NOX-independent mechanisms, but refer the reader to excellent reviews that discuss molecular mechanisms of NETosis in further detail (Papayannopoulos, 2018; Sollberger et al., 2018; Wigerblad and Kaplan, 2023).

NOX-dependent NETosis is activated by bacteria (Brinkmann et al., 2004), mitogenic stimuli (Amulic et al., 2017), cholesterol (Warnatsch et al., 2015) and antibodies (Behnen et al., 2014) via interaction with pattern recognition receptors (PRRs) on recipient neutrophils, which activate mitogen-activated protein kinase (MAPK) signalling and protein kinase C (PKC) (Chen et al., 2021; Hakkim et al., 2011). This leads to NOX activation and a cascade of chemical reactions that release reactive oxygen species (ROS), resulting in dissociation of the azurosome, which is a complex composed of eight proteins, three of which are serine proteases. Once released from the azurosome, these proteases migrate into the nucleus and promote nuclear membrane disintegration, chromatin decondensation and cytoplasmic membrane rupture, releasing NETs into the extracellular space (Metzler et al., 2014; Papayannopoulos et al., 2010). The less well understood NOX-independent pathway is thought to bypass the ROS production requirement via induction by calcium ionophores. During NETosis, histone H3 can undergo serine protease-dependent proteolysis (termed ‘clipping’), releasing histone H3 N-terminal tail peptides (Tilley et al., 2022). Histone clipping was initially suggested to be specific to NOX-dependent NETosis (Pieterse et al., 2018) but is now considered a distinguishing feature of all NETs and serves as a marker for their identification in cell culture and within tissues (Tilley et al., 2022). Another distinguishing feature of NETosis is activation of the peptidylarginine deiminase PADI4 (also known as PAD4), which citrullinates histones (Hagiwara et al., 2002).

The breakdown of cell and nuclear membranes and the release of chromatin to the extracellular space result in cell death. However, neutrophils have also been reported to perform chromatin extrusion in the absence of cell death. Human neutrophils, primed with granulocyte–macrophage colony-stimulating factor (GM-CSF) and stimulated with LPS, produce mitochondrial DNA NETs in a ROS-dependent manner and remain viable (Yousefi et al., 2009). Neutrophils infected with gram-positive *Staphylococcus aureus* display rapid NET release and develop diffuse and decondensed nuclei but do not undergo cell lysis (Pilsczek et al., 2010; Yipp et al., 2012). This process has been termed ‘vital NETosis’ (Yipp and Kubes, 2013). Despite these findings, understanding of vital NETosis is limited. Additional research is needed to delineate how vital NETosis occurs and why it may be advantageous. Speculatively, if surviving neutrophils retain some functionality (such as phagocytic capacity), then vital NETosis might serve to maximise and prolong the utility of these cells during immune responses. Indeed, there is evidence that anuclear human polymorphonuclear leukocytes retain their ability to kill *S. aureus* for a short time (Malawista and Van Blaricom, 1987; Malawista et al., 1989). It has also been shown that neutrophils that undergo vital NETosis retain their ability to crawl and contain granules, suggesting that they might maintain the ability to kill pathogens through conventional mechanisms (Yipp et al., 2012).

### Apoptosis and necrosis

Controlled cell death, or apoptosis, is an energy-dependent process crucial for development and homeostasis (reviewed in Elmore, 2007). Apoptosis involves chromatin condensation (pyknosis) and cell shrinking, followed by membrane blebbing and release of cell fragments known as apoptotic bodies (Häcker, 2000). This results in the release of fragmented DNA and histones into the extracellular space. Histones can also be found on the surface of immune cells (Watson et al., 1995), cerebellar neurons (Bolton and Perry, 1997), Schwann cells (Mishra et al., 2010) and microglia (Klein et al., 2014) in response to stress, where they have been described as an early marker for apoptosis (Gabler et al., 2004). Finally, nuclear expulsion and release of extracellular DNA–protein complexes has been identified as an apoptosis-induced phenotype in cancer cells (Park et al., 2023).

Histones and DNA can also be released during uncontrolled cell death (necrosis or oncosis). This energy-independent pathway involves cell and organelle swelling, an increase in membrane permeability, and chromatin clumping (Majno and Joris, 1995), after which membrane integrity is compromised and cellular contents (including chromatin) leak into the extracellular space.

### Active chromatin or histone release and secretion by cancer cells

Mounting evidence suggests that histones are actively secreted by cancer cells through conserved processes that do not result in cell death. Histones and nucleosomal DNA have been found to be the most abundant molecules in amphisomes (Jeppesen et al., 2019), a type of vesicle secreted from cells during autophagy. Furthermore, histones are highly abundant in exomeres, which are non-membranous nanoparticles that are actively trafficked out of multiple cancer cell lines (Zhang et al., 2018). It is unknown how histone secretion from cancer cells is stimulated or whether it confers antimicrobial protection. It is possible that it is aberrantly activated during deregulation of histone mRNA transcription and/or translation. Further experimentation is necessary to understand whether secreted histones impact cell transformation or the communication between cancer cells and the surrounding microenvironment.

### Extranuclear histones in lipid droplets

Lipid droplets are essential cell organelles with roles in lipid metabolism and cholesterol homeostasis. Histones H2A, H2Av and H2B have been identified in lipid droplets isolated from *Drosophila* embryos, among enzymes involved in lipid metabolism, signalling molecules and membrane trafficking proteins (Cermelli et al., 2006). A significant proportion of total embryonic histone H2A and H2B is found in such lipid droplets, suggesting that these organelles act as histone reservoirs during embryonic development (Cermelli et al., 2006). Because excess histones are cytotoxic, sequestering them in lipid droplet reservoirs until required could prevent both cellular damage and histone aggregation. Histones can transfer from lipid droplets to nuclei, supporting the theory that they are stored in lipid droplets during oogenesis (Cermelli et al., 2006; Johnson et al., 2018). Histones interact with lipid droplets via the docking protein Jabba (Li et al., 2012; Stephenson et al., 2021). Although conservation of this phenomenon across species is uncertain, histones H3 and H4 have been observed within mouse oocyte lipid droplets (Kan et al., 2012) and histone H3 has been found within lipid-containing microvesicles secreted from somatic cells (namely sebocytes; Nagai et al., 2005).

## Physiological and pathophysiological functions of extranuclear chromatin components

Chromatin released into the extracellular space undergoes fragmentation into (mono)nucleosomes, individual histones and DNA by proteases and deoxyribonucleases. These extracellular chromatin components impact animal physiology and pathophysiology through amplification of downstream inflammatory signalling responses, blood coagulation pathways and interactions with the extracellular matrix.

### Inflammation, tissue damage and repair

High concentrations of extracellular histones are cytotoxic due to their ability to bind and perforate plasma membranes and stimulate Ca^2+^ influx (Abrams et al., 2013; Collier et al., 2019; Ganapathy and Shyamala Devi, 2005; Silvestre-Roig et al., 2019; Xu et al., 2009). Elevated levels of extracellular histones have been observed in clinical conditions including sepsis, trauma, ischaemic stroke and autoimmune diseases (Abrams et al., 2013; Allam et al., 2012; Kawai et al., 2016; Silk et al., 2017; Xu et al., 2009). Anti-histone H3 and anti-histone H4 neutralising antibodies rescue typically lethal doses of LPS and tumour necrosis factor (TNF) in mouse sepsis models, highlighting histones as major players in disease progression (Xu et al., 2009). In a degenerating rat brain model, damaged brain tissue has been found to release histone H1, resulting in cortical neuronal death (Gilthorpe et al., 2013). Additionally, hyperacetylated histone H3.3 is resistant to proteasomal degradation and accumulates in the lungs of individuals with chronic obstructive pulmonary disease (COPD), where it promotes cell death, releasing more histones and creating an inflammatory positive feedback loop (Barrero et al., 2013). These findings have increased interest in therapeutics that target circulating histones. Small polyanions, which interact electrostatically with and neutralise cationic histones, have been shown to alleviate sepsis, deep vein thrombosis and cardiac and ischaemia injury in *in vivo* models (O'Meara et al., 2020; Xu et al., 2009).

Although hyperinflammation associated with chromatin release can lead to severe tissue damage, some evidence suggests that extracellular chromatin also has a role in wound healing and tissue repair (Sabbatini et al., 2021; Zhu et al., 2021). This reflects a broader emerging picture whereby inflammatory mediators may act as pro- or anti-repair signals in damaged tissues (Hausmann et al., 2024). The positive effects of extracellular chromatin are thought to be due to its antimicrobial properties but might be more far-reaching (Arampatzioglou et al., 2018). At sublethal concentrations or when complexed with DNA, histones function as damage-associated molecular patterns (DAMPs) (Nofi et al., 2022; Silk et al., 2017), which are endogenous danger signals exposed when cells die in response to tissue damage (Kono and Rock, 2008; Tang et al., 2012). PRRs (discussed below) sense DAMPs and alert neighbouring and immune cells, stimulating an innate inflammatory response (Huang et al., 2011; Li et al., 2022; Marsman et al., 2017; Richards et al., 2023; Saffarzadeh et al., 2012; Xu et al., 2011) and promoting tissue repair but also causing cytotoxicity and exacerbating tissue damage (Kono and Rock, 2008). PRR-dependent DAMP-associated signalling has been shown to promote recovery from acute lung injury (Jiang et al., 2005) and have a role in early skin wound healing (Chen et al., 2013). NETs specifically can promote skin wound healing by activating keratinocyte proliferation (Tonello et al., 2017). However, another study of skin wounding has shown that although NET-producing neutrophils are recruited to wound sites by pro-regenerative cues, NETs impede wound healing (Wier et al., 2021). NETs in the skin have also been shown to increase expression of connective tissue growth factors, production of collagen, and fibroblast differentiation, proliferation and migration (Chrysanthopoulou et al., 2014), which are effects that can support tissue repair but also lead to fibrosis.

The balance between pro-damage and pro-survival or pro-repair effects of extracellular chromatin might lie in the amounts of chromatin present. The pro-repair effect of NETs in skin has been shown to be concentration dependent, with low levels of NETs increasing keratinocyte proliferation but high levels having the opposite effect (Tonello et al., 2017). This broadly agrees with findings in endothelial cells, where treatment with extracellular histone concentrations under 50 µg/ml promotes autophagy via sestrin2 upregulation and decreased activation of AKT signalling, whereas concentrations exceeding 50 µg/ml induce p53 (TP53)–Bax-dependent apoptosis (Ibañez-Cabellos et al., 2018).

It is not understood whether the chromatin component of NETs or the presence of other associated factors determines the effects on tissue repair. The specific contributions of DNA, histones, certain histone PTMs or even histone-derived peptides are also unclear. The relative contributions of these factors have been difficult to ascertain due to the immense complexity of tissue architecture, cell death, inflammation, and the interplay between the cells of the damaged tissue, the tissue stroma and the immune system. Controlled studies of the contributions of these different components are needed to illuminate the role of extracellular chromatin in this process and could reveal therapeutic interventions that promote tissue repair.

### Cancer metastasis

The role of extracellular chromatin in cancer is also not entirely clear. The probable anti-tumour effects of NETs are typically associated with their ability to directly kill tumour cells (Arelaki et al., 2016; Millrud et al., 2017). However, increasing evidence shows that NET chromatin can promote cancer progression. Nucleosomal DNA from NETs functions as a scaffold that allows neutrophil elastase and matrix metalloproteinase 9 (MMP9) to cleave and remodel laminin, activating integrin signalling and promoting reactivation of dormant cancer cells (Albrengues et al., 2018). NETs can also capture and immobilise circulating cancer cells through interactions with β1 integrins on the cancer cell surface, promoting metastasis (Cools-Lartigue et al., 2013; Najmeh et al., 2017). Finally, extracellular DNA and chromatin-associated proteins can act as metastasis-promoting signals to cell surface receptors, as discussed below.

### Atherosclerosis and thrombosis

Extracellular chromatin exacerbates vessel occlusion disorders, such as deep vein thrombosis and atherosclerosis, through a variety of mechanisms (Martinod and Wagner, 2014; Sollberger et al., 2018). During infection or sterile inflammation, NETs formed within vessels stimulate and provide a scaffold for thrombus formation (Fuchs et al., 2010). Additionally, purified histones can activate platelets and promote blood coagulation (Fuchs et al., 2010; Vulliamy et al., 2019), and have been shown to induce fatal thrombocytopaenia in mice (Fuchs et al., 2011). Here, extracellular histones promote platelet aggregation by inducing Ca^2+^ influx and recruitment of adhesion proteins. Finally, extracellular histones can directly increase thrombin generation in the plasma by interfering with the activation of the natural anticoagulant protein C (PROC) (Ammollo et al., 2011). A recent study has shown that NETs released systemically after ischaemic stroke and myocardial ischaemia trigger extensive lymphocyte death within intestinal tissue (Tuz et al., 2024). The authors suggest that NET-generated thrombi restrict nutrients and oxygen to Peyer's patches (lymphoid tissues found in the intestines), resulting in altered metabolism and lymphocyte cell death.

## Sensing of extranuclear and extracellular chromatin

**Fig. 3.**
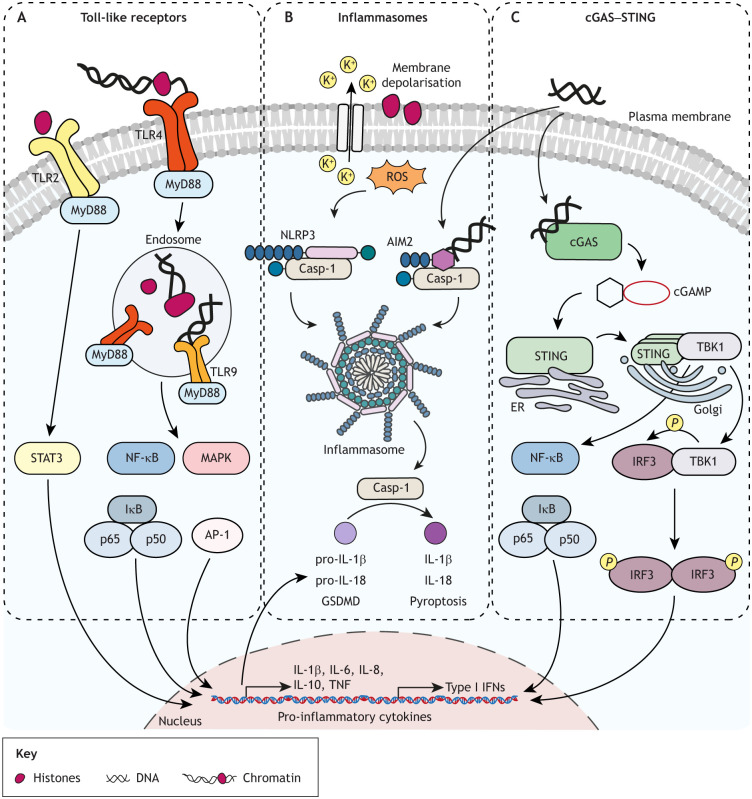
**Mechanisms of extracellular chromatin-mediated signalling.** (A) Toll-like receptors: extracellular histones and chromatin activate TLR2 at the cell surface and TLR4 on the cell membrane and in endosomes; endocytosed dsDNA is also recognised by endosomal TLR9. TLR2, TLR4 and TLR9 all signal via the adapter protein MyD88 and induce STAT3, NF-κB and MAPK signalling to stimulate production of type I interferons (IFNs) and pro-inflammatory cytokines (Akira and Takeda, 2004; Lin et al., 2010; Medzhitov et al., 1998; Park et al., 2013; Wilson et al., 2022). (B) Inflammasomes: AIM2 senses cytosolic dsDNA from host and pathogens in a sequence- and structure-unspecific manner (Fernandes-Alnemri et al., 2009; Hornung et al., 2009). Extracellular histones also depolarise the plasma membrane, causing intracellular oxidative stress, which activates NLRP3 (Beltrán-García et al., 2022; Muñoz-Planillo et al., 2013; Yu et al., 2024). NLRP3 and AIM2 oligomerise and form a scaffold for activation of caspase-1 (Casp-1), which subsequently cleaves pro-inflammatory cytokines of the IL-1 family into their bioactive forms (IL-1β and IL-18), inducing inflammation, and further mediates a form of gasdermin D (GSDMD)-mediated programmed cell death (pyroptosis) (Martinon et al., 2002; Shi et al., 2015). (C) cGAS–STING: binding of self-DNA to cGAS triggers synthesis of the second messenger molecule 2′,3′-cyclic GMP–AMP (cGAMP). cGAMP subsequently binds to the adapter protein STING (also known as TMEM173) in the endoplasmic reticulum (ER), leading to its dimerisation and activation. STING dimers translocate to the Golgi, where they oligomerise to recruit and activate TANK-binding kinase 1 (TBK1). TBK1 phosphorylates the transcription factor IRF3, causing its translocation to the nucleus, stimulation of type I IFN (IFNα and IFNβ) expression and subsequent transcription of IFN-stimulated genes. STING also serves as a scaffold for activation of IKKε (also known as IKBKE), resulting in nuclear translocation of NF-κB and production of the pro-inflammatory cytokines TNF, IL-6 and IL-1β (Ishikawa et al., 2009; Sun et al., 2013; reviewed in Decout et al., 2021; Skopelja-Gardner et al., 2022). AP-1, activator protein 1; IκB, inhibitor of NF-κB protein; p50, NF-κB p50 subunit, encoded by *NFKB1*; p65, NF-κB p65 subunit, encoded by *RELA*.

Two main routes for sensing extracellular chromatin components have been described: (1) via endocytosis and sensing within endosomes and the cytosol, and (2) via binding to cell surface receptors and activation of downstream signalling (Fig. 3).

### cGAS–STING

Cytosolic self-DNA acts as a DAMP that activates the cyclic GMP–AMP synthase (cGAS)–stimulator of interferon genes (STING) pathway. This induces an inflammatory response through nuclear translocation of the transcription factors NF-κB and interferon regulatory factor 3 (IRF3), and stimulation of expression of pro-inflammatory cytokines and type I interferons (IFNα and IFNβ) (Ablasser and Chen, 2019; Ishikawa et al., 2009) (Fig. 3C).

The cGAS–STING pathway is active in immune cells (macrophages, dendritic cells and T cells) as well as non-immune cells (epithelial cells and fibroblasts). Activation of this pathway has been shown to exacerbate inflammation in fibrotic interstitial lung disease (Benmerzoug et al., 2018) and promote autoimmunity, for example in systemic lupus erythematosus (SLE) and Aicardi–Goutières syndrome (Gao et al., 2015; Skopelja-Gardner et al., 2022). cGAS–STING activation also promotes cellular senescence in a non-cell-autonomous manner by regulating the senescence-associated secretory phenotype (SASP) (Glück et al., 2017). Cancer cells can hijack or dysregulate this pathway to evade immune responses, promote tumour growth or shape the tumour microenvironment (Marcus et al., 2018; Schadt et al., 2019), which can impede oncolytic viral therapy (Arwert et al., 2020). However, recent research has revealed that cGAS is a predominantly nuclear protein that is tightly tethered to chromatin and locked in its inactive state through high-affinity binding to nucleosomal histones, challenging the prevailing view of cGAS as a cytosolic DNA sensor (Boyer et al., 2020; Cao et al., 2020; Gentili et al., 2019; Kujirai et al., 2020; Michalski et al., 2020; Orzalli et al., 2015; Pathare et al., 2020; Volkman et al., 2019; Zhao et al., 2020). The relative contributions of cytosolic and nuclear cGAS to cGAS–STING activation in response to aberrant double-stranded DNA (dsDNA) are a matter of active investigation, as are the signals and molecular mechanisms that release cGAS from nucleosome sequestration (de Oliveira Mann and Hopfner, 2021). In cancer cells, the DNA double-strand break sensor MRE11 can untether nuclear cGAS from nucleosomes, enabling cGAS activation by dsDNA and suppression of tumorigenesis (Cho et al., 2024).

### CCDC25

The transmembrane coiled-coil domain containing protein 25 (CCDC25) was identified as a cancer cell surface receptor for extracellular DNA (Yang et al., 2020). Upon binding of NET DNA, CCDC25 activates the ILK–β-parvin pathway, enhancing cell motility and promoting cancer metastasis (Yang et al., 2020). CCDC25 is additionally expressed in human umbilical vein endothelial cells and impacts the progression of gastric cancer by activating AKT–mTOR signalling and promoting angiogenesis (Yang et al., 2023). The specific interaction between CCDC25 and NET DNA has been leveraged to create biomimetic CCDC25-overexpressing cell membrane hybrid liposomes, which have been successfully used to inhibit colorectal cancer liver metastases (Wang et al., 2023).

CCDC25 has also been implicated in allergic inflammation and asthma progression (Lu et al., 2021). DNA from eosinophil extracellular traps (EETs) in the bronchoalveolar fluid binds to CCDC25 on pulmonary neuroendocrine cells (PNECs). This activates PNECs via an ILK–PKCα–CRTC1 pathway and induces secretion of neuroendocrine factors that amplify the allergic asthma response (Lu et al., 2021).

Little is known about the regulation of CCDC25, but cholesterol biosynthesis has been shown to promote its expression in a lipid raft-dependent manner (Tang et al., 2022). Publicly available transcriptomic data indicate that CCDC25 is expressed in various tissues under homeostatic conditions (Uhlén et al., 2015), suggesting that there is potentially more to be discovered about extracellular DNA sensing in homeostasis.

### Inflammasomes

Inflammasomes are large cytosolic multiprotein complexes assembled by PRRs following the detection of infection- or stress-associated stimuli. Several families of intracellular PRRs are components of inflammasome complexes, including the nucleotide-binding oligomerisation domain, leucine-rich-repeat-containing protein (NLR) family member NLRP3 and the protein absent in melanoma 2 (AIM2), which are activated by chromatin DAMPs (Broz and Dixit, 2016) (Fig. 3B). Activation of the AIM2 inflammasome contributes to normal brain development by eliminating unfit neurons through GSDMD-mediated pyroptosis (Lammert et al., 2020). AIM2 deficiency causes retention of damaged neuronal cells and is associated with anxiety-related behaviours in mice (Lammert et al., 2020). However, the AIM2 inflammasome is detrimental in ischaemic brain injury and subarachnoid haemorrhage (Yuan et al., 2020; Zhang et al., 2020), and its activation induces pyroptosis in primary cortical neurons during traumatic brain injury (Adamczak et al., 2014). Beyond the brain, reduced AIM2 levels correlate with prostate and colorectal cancer development (Dihlmann et al., 2014; Ponomareva et al., 2013), and increased AIM2 expression is associated with SLE (Javierre et al., 2010) and psoriasis (Dombrowski et al., 2011).

Oxidative stress also influences extracellular chromatin-mediated inflammasome activation. Extracellular histones cause plasma membrane depolarisation, increasing intracellular oxidative stress and subsequently activating NLRP3 for inflammasome formation (Allam et al., 2013; Beltrán-García et al., 2022). Both histone hyperacetylation and antioxidants have been found to play a protective role against NLRP3-mediated inflammation (Beltrán-García et al., 2022). Extracellular histones also activate the NLRP3 inflammasome through Toll-like receptor 9 (TLR9)-dependent generation of ROS after ischaemia–reperfusion (I–R) injury (Huang et al., 2013).

Exacerbated NLRP3 inflammasome activation contributes to multiple inflammatory diseases, including peritonitis (Allam et al., 2013) and sepsis (Beltrán-García et al., 2022). NLRP3 deficiency protects against renal I–R injury (Iyer et al., 2009), and NLRP3 inflammasome inhibition limits infarct size following myocardial I–R (Toldo et al., 2016), revealing the NLRP3 inflammasome as a major mediator of sterile inflammation and organ damage following necrotic cell death and tissue injury. However, inflammasome activity has also been suggested to mediate tissue regeneration and wound healing via secretion of IL-1β and IL-18 cytokines (Artlett, 2013; Rathinam and Chan, 2018).

### Toll-like receptors

Toll-like receptors (TLRs) are a well-known family of PRRs responsible for initiating an innate immune response. They alert the immune system to the presence of pathogens upon sensing pathogen-associated molecular patterns (PAMPs), leading to NF-κB- and MAPK-mediated transcription of pro-inflammatory cytokines (such as TNF and IL-6), chemokines and effector molecules, depending on the activated cell type (Fitzgerald and Kagan, 2020). Nucleic acids and histones also activate members of the TLR family, acting as host-derived DAMPs (Fig. 3A). TLR2, which is expressed on the plasma membrane, and TLR4, which is localised both on the plasma membrane and in endosomal vesicles (Allam et al., 2012; Tsourouktsoglou et al., 2020; Xu et al., 2011), bind histones and nucleosomes in addition to recognising bacterial lipoproteins and LPS, respectively. Extracellular histone-induced TLR2 and TLR4 signalling leads to pro-inflammatory responses with high levels of the inflammatory cytokines TNF, IL-6 and IL-10, inducing tissue injury and organ failure (Xu et al., 2011). TLR2- and TLR4-knockout mice are protected against histone-induced fatal liver injury (Xu et al., 2011). Similarly, TLR2- and TLR4-mediated signalling via MyD88, NF-κB and MAPKs causes extracellular histone-mediated acute kidney injury (Allam et al., 2012), platelet activation and thrombosis (Semeraro et al., 2011). In retinal detachment, histones released into the eye vitreous body induce pro-inflammatory IL-8 (also known as CXCL8) via TLR4 and the ERK1/2 and p38 MAPK pathways (Kawano et al., 2014). Extracellular histones further promote hepatocellular carcinoma cell migration and invasion through TLR4-, ERK1/2- and NF-κB-mediated production of chemokines such as C-C motif ligand 9 (CCL9, also known as CCF18) (Chen et al., 2016a). Notably, NET-derived extracellular histones have been shown to promote pro-inflammatory T helper 17 (Th17) cell differentiation via TLR2 and STAT3 signalling (Wilson et al., 2022), providing evidence of their role as cell modulatory molecules that impact cell fate changes beyond acute inflammation.

TLR9 is expressed in endosomes and recognises endocytosed extracellular single-stranded DNA containing unmethylated cytosine–phosphate–guanosine (CpG) motifs (Bauer et al., 2001; Hemmi et al., 2000). CpG motifs in mammalian DNA are mostly methylated; hence, the response of TLR9 to self nucleic acids is limited (Lind et al., 2022). Nevertheless, TLR9 is linked to autoimmune and autoinflammatory disorders including SLE and psoriasis, and it has been demonstrated that mammalian DNA can stimulate TLR9 when present in immune complexes (Viglianti et al., 2003). TLR9 is also expressed in tumour epithelial and stromal cells across different cancer types, and synthetic TLR9 ligands can stimulate invasion (Ilvesaro et al., 2008). DAMPs released into the tumour microenvironment after radiation therapy trigger TLR9 activation in myeloid cells, promoting angiogenesis and tumour recurrence (Gao et al., 2013). TLR9 has additionally been identified as a key molecule in long-term memory formation (Jovasevic et al., 2024): in a mouse model of contextual fear conditioning, a subset of hippocampal neurons undergoes persistent DNA damage upon memory acquisition, triggering the release of nuclear DNA, which is recognised by TLR9, initiating the NF-κB-dependent inflammatory signalling essential for memory persistence.

### Synergy of extracellular DNA and histones in TLR signalling

At low concentrations, histones induce pro-inflammatory cytokine production, but at high concentrations they are cytotoxic and kill cells before cytokines are produced. How is signalling below the cytotoxicity threshold achieved? A study in human monocytes has revealed that synergy between histones and DNA is critical for sublethal signalling: histones activate TLR4, whereas DNA recruits TLR4 to histone-containing endosomes, achieving induction of pro-inflammatory cytokines only in synergy (Tsourouktsoglou et al., 2020). In the absence of DNA, TLR4 does not translocate to histone-containing endosomes. This synergistic effect has also been reported in fatal liver injury, where extracellular DNA further increases histone-induced TLR2 and TLR4 signalling (Xu et al., 2011).

### Receptor for advanced glycation end products

Chromatin-associated factors such as HMGB1 and S100 proteins are released into the extracellular space upon cell death or NETosis. These factors enhance DAMP-mediated inflammatory signalling via TLRs and the receptor for advanced glycation end products (RAGE, also known as AGER) (reviewed in Hu et al., 2023). HMGB1–DNA complexes released from damaged cells activate both TLR9 and RAGE, which co-operate to stimulate plasmacytoid dendritic cells and B cells (Tian et al., 2007). By sensing extracellular DNA and facilitating its uptake into endosomes, RAGE further promotes TLR9 activation and inflammatory signalling (Sirois et al., 2013). Extracellular histones have recently been identified to bind RAGE and become internalised in cells that do not normally perform phagocytosis (Yang et al., 2022).

### Endogenous neutralisers of extracellular chromatin

Several endogenous molecules, including factor VII-activating protease (FSAP, also known as HABP2), the extracellular chaperone clusterin (CLU), soluble carcinoembryonic antigen-related cell adhesion molecule (CEACAM) 8 and heparin, can neutralise extracellular histones and/or suppress histone-induced cytotoxicity and pro-inflammatory signalling. FSAP is activated by and proteolyses histones, protecting against histone-mediated cytotoxicity in inflammatory conditions such as sepsis (Marsman et al., 2017). CLU binds circulating histones and promotes their clearance via receptor-mediated endocytosis (Augusto et al., 2023; Cunin et al., 2016; Patel et al., 2023). Extracellular chromatin triggers neutrophil secretion of CEACAM8 (Ribon et al., 2019), which binds CEACAM1 and inhibits TLR2-triggered immune responses (Singer et al., 2014). Thus, CEACAM8 secretion represents an indirect mechanism of neutralisation of histone-mediated DAMP signalling. Binding of heparin to extracellular histones abolishes their platelet-activating potential, preventing thrombosis (Semeraro et al., 2011) and protecting mice from histone-induced thrombocytopaenia, tissue damage and death (Fuchs et al., 2011). Of note, heparin and histones are both evolutionarily conserved. Hence, heparin binding could serve as an innate histone neutralisation mechanism that reduces collateral tissue damage (Fuchs et al., 2011).

## Post-translational modifications associated with extracellular histones

The mechanisms mediating the eviction of histones from the nucleus, their trafficking through the cell and/or their downstream functions are incompletely understood. Histone PTMs are possible modulators of these processes. It is also plausible that histones, or even DNA, are subject to different location- or environment-dependent chemical modifications. Notably, non-enzymatic or non-reversible histone PTMs are difficult to reconcile with the dynamic regulation required for gene transcription. It is possible that there is less need for reversal of PTMs on histones that have been evicted from the nucleus, or that certain PTMs mark histones for release or modulate their bioactivity. Below, we summarise current knowledge on PTMs associated with extracellular histones.

### Citrullination

Citrullination, the non-reversible conversion of arginine to citrulline, is almost synonymous with extracellular chromatin and is often used as a marker of NETosis and nuclear expulsion (Park et al., 2023; Sollberger et al., 2018). Histone citrullination has been reported as a key late step in NETosis required for the dissociation of histones from DNA and their subsequent release (Chen et al., 2016b; Thiam et al., 2020). However, whether citrullination is a mediator of NETosis, or merely a consequence of this process, is significantly debated (Kenny et al., 2017). If NETosis can proceed without citrullination, this begs the question of what the role of this modification is. An elegant study has demonstrated that, rather than mediating the execution of NETosis, citrullination potentiates downstream TLR4-mediated inflammatory signalling (Tsourouktsoglou et al., 2020). However, contrary to what might be expected from the association between NETosis and antimicrobial immunity (Brinkmann et al., 2004), citrullination of extracellular histone H3 reduces its antibacterial activity but enhances its proteolysis by neutrophil elastase (Tanner et al., 2021). The authors suggest that citrullination helps resolve acute inflammation after infection – a hypothesis supported by an *in vivo* study showing that citrullination reduces histone bactericidal activity in infection-induced NETs (Li et al., 2010). An independent study has shown that although extracellular histone H4 induces NETosis through neutrophil cell membrane permeabilisation and Ca^2+^ influx, these processes are dampened by histone H4 citrullination (Shi et al., 2021). Thus, citrullination can dampen the inflammatory and pathological effects of NETs. These results are perplexing in light of the strong association between citrullination and autoimmunity (Ghari et al., 2016; Kawalkowska et al., 2016; Knight et al., 2015).

It is interesting to consider that, although the ability to carry out ETosis is a general feature of multicellular organisms, citrullination emerged in animals relatively late in evolution (Cummings et al., 2022). Although ETosis can occur without citrullination, it is possible that citrullination has been co-opted to regulate and fine-tune extracellular chromatin functions.

### Acetylation, carbamylation, proteolytic cleavage and other PTMs

Some evidence exists that acetylation, a modification abundant on nuclear histones, enhances the immune-stimulatory potential of NETs (Chapman et al., 2019; Rother et al., 2017). Carbamylation, or homocitrullination, is also present on NET histones and is the source of rheumatoid arthritis-specific autoantigens (O'Neil et al., 2020). Proteomic analyses of NET histones have further identified methionine oxidation (sulfoxide), formylation, thiol alkylation, and deamination of asparagine and glutamine residues (Petretto et al., 2019). However, it is unclear whether these PTMs occur specifically on extracellular histones, at which point during the NETosis process they might take place and whether they modulate downstream NET functions.

Proteolytic cleavage, even in the absence of chemical modifications, is increasingly regarded as a type of PTM, as it generates new N- or C-termini that may have different binding specificities or biological activities (Rogers and Overall, 2013). This is particularly pertinent in the context of extracellular chromatin, which is accompanied by the secretion of proteolytic enzymes and histone clipping (Tilley et al., 2022). When considering extracellular histones as potentially bioactive molecules, it is important to also consider their proteolytic products.

Theoretically, DNA could also be modified during release from the nucleus, although this is made less likely by the fact that there are only a handful of known DNA modifications, which are catalysed by predominantly nuclear enzymes. However, oxidatively damaged DNA has been observed in SLE (Kurien and Scofield, 2008) and has been shown to contribute to tissue inflammation (Tumurkhuu et al., 2020), suggesting that, at least in some instances, the high levels of oxidative damage that accompany extracellular chromatin release might result in DNA modifications.

## Extranuclear and extracellular histones as signals – beyond immune defence?

Though the nuclear and non-nuclear functions of histones are typically studied in relative isolation within the epigenetics and immunology fields, respectively, cell biology is a continuum. Some of these functions are therefore likely to be executed in parallel and might impact each other.

Extracellular chromatin and histones have far-reaching implications in inflammatory disease, cancer and injury. However, a picture is also emerging of their potential biomodulatory roles beyond acute induction of inflammation. Findings such as the role of extranuclear histone H1.2 as a mediator of apoptosis (Konishi et al., 2003), the ability of extracellular histones to modulate T cell differentiation outcomes through TLR2 signalling (Wilson et al., 2022), the perplexing presence of extranuclear subH2B in sperm (Raman et al., 2022), and the metabolic and catalytic functions of histones, along with the discovery of mechanisms for the active release of histones from both dying and living cells, give us food for thought.

It is exciting to theorise that extranuclear and extracellular histones or chromatin might have new and previously unpredicted functions as bioactive molecules. The fact that different histone proteins operate as antimicrobial agents in the extracellular space of different tissues is perplexing, given that histone genes are constitutively and highly expressed in all cells. This could suggest a more sophisticated set of functions, whereby different histone proteins induce downstream cellular responses depending, for example, on the types of cell surface receptors expressed in certain tissues.

Within a plethora of known and as-yet-undiscovered histone PTMs, modifications specific to extranuclear and extracellular chromatin might exist, and new biological functions might be ascribed to known PTMs in an extranuclear context. For example, PTMs, or indeed the primary sequence of the different histone proteins, might differentially affect histone binding affinity to PRRs and fine-tune the outcomes of downstream signalling. It also remains to be revealed whether binding to PRRs occurs through the histone tail or globular domain, or whether histone proteolysis produces PRR ligands. Future mutagenesis studies could ascertain the contributions of different histone sequences and their post-translationally modified versions.

Finally, the continuous evolution of histone variants expressed in certain organismal and tissue contexts could indicate not only new roles in genome organisation and regulation, but also functions that go beyond these classical capabilities. Experiments involving the careful titration of native or post-translationally modified histones, or modulation of histone-binding receptors, will present new challenges and opportunities for exploration of the underlying mechanisms and biological outcomes of histone externalisation.
